# Purification, characterization, and identification of 3‐hydroxy‐4‐methoxy benzal acrolein–an intermediate of synthesizing advantame

**DOI:** 10.1002/fsn3.1307

**Published:** 2020-01-01

**Authors:** Bo‐Ru Chen, Qiang Liu, Huan Wang, Zi‐Yuan Gao, Azhari Siddeeg, Si‐Ming Zhu

**Affiliations:** ^1^ School of Food Science and Engineering South China University of Technology Guangzhou China

**Keywords:** 3‐hydroxy‐4‐methoxy benzal acrolein, HPLC, low‐pressure column chromatography, purification

## Abstract

Advantame is a novel sweetener, and 3‐hydroxy‐4‐methoxy benzal acrolein is an important intermediate to synthesize the sweetener. The aim of this study was to evaluate a new low‐cost method to purify 3‐hydroxy‐4‐methoxy benzal acrolein, and the crude intermediate was used as raw material. The intermediate was purified using a low‐pressure column chromatography with a C_18_ column, using a methanol‐water (6:4, v/v) at a flow rate of 6.0 ml/min, and the loading amount of the crude intermediate in solution was 10.0 mg in total. A method for the analysis of 3‐hydroxy‐4‐methoxy benzal acrolein was established using high‐performance liquid chromatography (HPLC). This method was validated in terms of its linearity, repeatability, accuracy, detection limit, and quantitation limit. The calibration curves of 3‐hydroxy‐4‐methoxy benzal acrolein were linear (*r* > .999) over a wide concentration range of 0.005–0.08 mg/ml, by comparing the ratio of signal to noise, and the detection limit was 5.0 ng/ml and the quantification limit was 15.0 ng/ml. Good repeatability was obtained (RSD < 2%, *n* = 6), and the recoveries calculated using mixed sample previously quantified ranged from 94.5% to 106.37%. As long as, this method has been successfully applied to the analysis of 3‐hydroxy‐4‐methoxy benzal acrolein; therefore, the method can be put into practical use during the industrial synthesis and real‐time detection of the intermediate.

## Graphical abstract

In this study, a new low‐cost method for the preparation and purification of a sweetner intermediate named 3‐hydroxy‐4‐methoxy benzal acrolein was described. The purifying method and analysis method could be a reference to develop other sweetener intermediates.

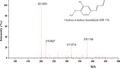

## 1 INTRODUCTION

Advantame is an N‐substituted (aspartic acid portion) derivative of the sweetener of aspartame and is one of the newest additives in the group of low‐calorie, high‐potency sweetening agents. The sweetener has been approved for human consumption by the U.S.FDA (2014) recently (Kux, 2014). The sweetness of advantame is approximately 20,000 times that of sucrose (Renwick, 2011). The high intensive sweetness of advantame provides a potential for being used as a sweetener in various food products at a content level far below that of sucrose or other high‐intensity sweeteners. Advantame has been demonstrated as a good sweetener in iced tea, coffee (warm and hot), powdered beverage formulations, etc. And as a flavor enhancer in yogurt, advantame can be used in beverages or chewing gum, etc. In view of its excellent characteristics and broad market prospects, it is of great economic value and academic value to study the synthesis of advantame (Otabe Fujieda, & Masuyama, 2011a, 2011b). The method of synthesizing advantame was usually based on cinnamaldehyde derivatives, and aspartame was obtained from the ring‐opening reaction and hydrogenation reaction of the derivatives or intermediate (Kawahara, Nagashima, & Takemoto, 2001). Therefore, cinnamaldehyde derivatives are the key intermediate to the production or preparation of advantame (Gan, Yan, & Li, 2011). Advantame was usually synthesized through three steps of reaction using cinnamic acid as original beginning substrate. Nevertheless, its industrial application was limited for its tedious preparation process and the usage of poisonous phosgene (Fujita, Funakoshi, Mori, & Takemoto, 2009). To short or simplify the preparation process, in the present study, benzaldehyde derivatives containing hydroxyl electron‐donating groups or methoxy electron‐donating groups were selected to react with aldehyde taking place aldol reaction, the advantame intermediate was obtained from the aldol reaction, the intermediate was obtained from the aldol reaction, and then, advantame was prepared through a ring‐opening and hydrogenation reaction on the basis of the intermediate at the same time, so advantame was prepared in just two simple steps (Wu & Zheng, 2009). To our knowledge, a series of safety toxicology studies of advantame have been performed (Otabe, Fujieda, Masuyama, Ubukata, & Lee, 2011c; Sclafani & Ackroff, 2015; Ubukata, Nakayama, & Mihara, 2011), and a method using HPLC and LC‐MS/MS for the determination of advantame in food was described (Kobayashi, Terada, Nakajima, 2015). However, currently there is barely any detailed information on the analysis or detection method of the advantame intermediate, and even the reference substance of advantame intermediate cannot be bought from the market.

Accordingly, in this research, high purity advantame intermediate was obtained using low‐pressure column chromatography, the effects of mobile phase proportion, loading amount and the flow rate on separation efficiency was done, and these parameters were furtherly optimized using single factor experimental design. Therefore, a simple, specific, and sensitive method for determining the advantame intermediate was established, the method involved the extraction of the intermediate using methanol, and the separation of the intermediate via C_18_ column chromatography followed by the determination of the intermediate with high performance liquid chromatography (HPLC). Simple laboratory validation of the method was performed in terms of linearity, accuracy, and precision.

## 2 MATERIALS AND METHODS

### 2.1 Chemicals and reagents

Isovanillin was supplied by Shandong Moon Fairy Biological Technology Co., Ltd. Reference substance 3‐hydroxy‐4‐methoxy benzal acrolein was synthesized by us. Methanol of HPLC grade was acquired from Tianjin Kermel Chemical Reagent Co., Ltd. Acetaldehyde, hydrochloric acid, and NaOH of analytical grade were purchased from Guangzhou Chemical Reagent Co., Ltd. All others chemicals were of analytical grade.

### 2.2 Sample preparation

Isovanillin (12.17 g, 0.08 mol) and NaOH (32 g, 0.8 mol) were added into 200 ml distilled water; the whole system was mixed gently and then cooled down to −5°C; 28% (29 g, 0.184 mol) acetaldehyde was continuously dropped to the alkaline solution within 2 hr followed by 1h agitation after the last drop; and then, the reaction was stopped by 36% HCl (79.08 g, 0.78 mol), The crude product was obtained after the following procedure: The reactive solution was filtrated, and then, the redidual solid was dissolved again in 34 ml methanol at 60°C and evaporated in a certain vacunm (RE‐52E, Yarong biochemical instrument factory, Shanghai, China) to recover the solvent at 40°C; finally, the residual system was dried at 50°C in a vacuum dryer.

### 2.3 Preparation of reference substance using low‐pressure column chromatography

The crude product was purified using a EZ purifier Ⅱ preparative low‐pressure column chromatography (LiSui Technologies Co., Ltd.) with a 360 mm × 40 mm ODS tube. Compound separation was monitored using an absorbance detector at 341 nm. Binary mixed solvents of methanol and water were used as mobile phase at a steady flow rate. Crude product was resolubilized in 5 ml 50% methanol and filtered through a 0.45‐μm membrane and then injected into the C_18_ column. The solution corresponding to the first absorption peak in the elution curve was collected together, concentrated, freeze‐dried under reduced pressure, and weighed. The effect of eluent, flow rate, and loading amount on purification efficiency was investigated by changing the flow rate of 4, 6, 8, and 10 ml/min, loading amount of 6, 8, 10, and 12 ml (the sample concentration was 1 mg/ml), and volume ratio of methanol–water (v/v) as 4:6, 5:5, 6:4, and 7:3.

Normalization method of HPLC was adopted to evaluate the purity of the samples. The sample yield was the mass ratio of content of intermediates of samples after purification to before purification, and the spectroscopy methods were used to identify the structures of the chemical compounds.

### 2.4 UV‐Visible spectroscopy

3‐Hydroxy‐4‐methoxy benzal acrolein was dissolved in 50% methanol, to determine the maximum wavelength through the UV‐visible absorption spectrum scanning conducted on a TU‐1810 double‐beam spectrophotometer (Purkinje General Instrument Co., Ltd.) at room temperature (25 ± 3°C) by using 1 cm quartz cell. The spectra were recorded from 200 nm to 800 nm.

### 2.5 HPLC analysis

The purified intermediate was analyzed by a WUFENG HPLC apparatus equipped with a WUFENG LC‐P100Plus pump, a WUFENG LC‐UV100Plus diode array detector The detecting method was improved on the basis of available literature (Sait et al., 2015; Shen et al., 2016). The column was a Polaris C_18_‐A column (250 mm  × 4.6 mm, 5 μm; Agilent Technologies Co., Ltd.). The solvent system consisted of methanol and ultrapure water (6:4, v/v) with isocratic elution at a flow rate of 0.5 ml/min, column temperature was set at 35°C, the intermediate sample solution was filtered through 0.45‐μm filtration membrane, and 20 μl of the sample was injected, monitoring was performed at 341 nm. Accurately weighed 1 mg of intermediate reference substance and crude sample, respectively, and dissolved in methanol–water system (50:50, v/v) to obtain the stock solutions, then detection and analysis under the above chromatographic conditions.

### 2.6 FTIR spectroscopy

The FTIR spectra of the product or the intermediate were obtained by a VERTEX 33 infrared spectrometry (Bruker Optics) with a blank KBr disk as background, and the mixture of the sampled intermediate and KBr was dried and condensed into a transparent tablet for measurement. The spectrum was recorded within the range from 400 to 4,000/cm.

### 2.7 Mass spectroscopy analysis

Mass spectroscopy analysis was recorded on a Agilent1290 high‐resolution mass spectrometer (Bruker) with an electrospray ionization (ESI) interface. The intermediate was analyzed in positive‐ion mode, the effluent was infused into an electrospray ionization source, and the analysis was performed using the following acquisition parameters: ion capillary voltage, 3,500 V; desolvation temperature, 180°C; ion release delay, 5 ms; and nebulizer, 30 psi. Argon was the collision gas (collision energy 16 eV), and nitrogen was used as desolvation gas (dry gas flow, 4.0 L/min). Scanned mass spectra of the compounds were obtained from 50 up to 1,000 m/z.

### 2.8 NMR analysis

The purified intermediate was identified by ^1^H and ^13^C NMR spectrometry. The ^1^H and ^13^C NMR spectra were performed on an AVANCE III HD 400 spectrometer (Bruker Biospin Gmbh) operating at 400 MHz in MeOD_._

### 2.9 Method validation

#### 2.9.1 Precision test

The intraday was assessed by running six replications of the same sample for the peak area within one day (Bae, Jayaprakasha, Jifon, & Patil, 2012). Treat 0.03 mg/ml, 0.06 mg/ml of the intermediate sample under the described method in six replications, separately. The precision was represented as relative standard deviation (RSD%) (Ariffin, Ghazali, & Kavousi, 2014).

#### 2.9.2 Recovery test

The accuracy was assessed by implementing the recovery method. The recoveries method were calculated by the formula:$\text{recoveris}\;\left(\%\right)=\left[C_{\text{spike sample}}-C_{\text{unspike sample}}\right]*100$ (Yan et al., 2016). Five different concentrations of 0.08 ml of reference substance (0.005, 0.02, 0.04, 0.06, 0.08 mg/ml) were added to 0.02 ml of known content crude solution (0.02 mg/ml) at five repeatability.

#### 2.9.3 Calibration curve

A five‐point calibration curve was constructed by peak area versus reference substance concentration. To prepare the standard solution, 10 mg reference substance were dissolved in 50% methanol and the volume adjusted to 10 ml to obtain a stock solution of 1mg/ml, standard working solutions (0.005, 0.02, 0.04, 0.06, 0.08 mg/ml) were made by serial dilution of the stock solution with 50% methanol, the peak area as ordinate and the concentration as abscissas, draw standard curve and regression equations.

#### 2.9.4 Limit of detection and limit of quantification

LOD as the lowest concentration was obtained by the steady dilution of the standard solution until the ratio of signal to noise is 3:1 (De Beer and Joubert, 2010). LOQ is defined as the lowest concentration of a sample by diluting corresponding standard to obtain the ratio of signal to noise of 10:1 (Bonfatti, Grigoletto, Cecchinato, Gallo, & Carnier, 2008).

## 3 RESULTS AND DISCUSSION

### 3.1 Preparation and identification of reference substances 3‐hydroxy‐4‐methoxy benzal acrolein

#### 3.1.1 Technological optimization of preparative low‐pressure column chromatography

The flow rate was a significant factor. Experimental results (Table 1) showed that as the flow rate (4, 6, 8, 10 ml/min) increased, the retention or collection time shortened. However, the peaks were not completely separated while the flow rate was too fast to have plenty of time for separation, causing lower separation efficiency. Generally speaking, the lower the flow rate is, the more possibly a good separation can be obtained, because of the separation time is enough for the sample diffusion and elution in column (Rasmussen & Scherr, 1987). Nevertheless, taking time consumption and separation efficiency into consideration, the optimum flow rate was 6 ml/min.

**Table 1:** Purity and yield of 3‐hydroxy‐4‐methoxy benzal acrolein under different flow rate of mobile phase

Flow rate(mL/min)	3‐Hydroxy−4‐methoxy benzal acrolein		Retention time
	Purity(%)	Yield(%)	(min)
4	89.35 ± 0.29	27.76 ± 0.12	60.57 ± 1.34
6	95.31 ± 0.37	35.23 ± 0.22	48.15 ± 0.73
8	91.56 ± 0.39	33.28 ± 0.19	45.83 ± 1.04
10	84.67 ± 0.45	32.34 ± 0.26	42.59 ± 0.68
12	0	0	0

Note

The volume ratio of methanol‐water was 6:4 (v/v), the injected volume was 5 ml, sample concentration was 1 mg/ml

The loading amount was also a significant factor, a series of experimental results showed that with an increasing loading quantity (6, 8, 10, 12 mg), and the separation efficiency increased firstly and then decreased (Table 2). Generally speaking, the separation efficiency should decrease as the larger the loading amount was, the larger the pressure was. In addition, a large loading amount may cause the column being blocked. However, the blocking phenomenon was not observed at the loading amount of 6 mg in our research. As almost all impurities waste was fixed and eluted separately regardless of the loading amount within the loading amount ranging from 6 to 10 mg, relative impurities would decrease as the loading amount increased (Kuang, Liang, &Yuan, 2010). Thus, the separation efficiency was improved with the increasing loading amount ranging from 6 to 10 mg. However, the efficiency dropped at the loading amount of 12 mg or more. Possible reason for it may be that overloading amount leads to an overlap of nearby peaks and a downtrend in separation efficiency. Therefore, the loading amount was set as 10 mg.

**Table 2:** Purity and yield of 3‐hydroxy‐4‐methoxy benzal acrolein under different loading amounts

Loading amount(mg)	3‐Hydroxy−4‐methoxy benzal acrolein	
	Purity(%)	Yield(%)
6	96.27 ± 0.37	31.12 ± 0.22
8	95.83 ± 0.15	32.18 ± 0.18
10	94.31 ± 0.11	35.23 ± 0.12
12	52.34 ± 0.09	40.21 ± 0.32

Note

The volume ratio of methanol‐water was 6:4 (v/v), the flow rate was 6 ml/min.

The proportion of mobile phase plays an important role in column chromatography purification process. The determined purities of the intermediate 3‐hydroxy‐4‐methoxy benzal acrolein in a crude product was greatly different in three elution modes, since different elution modes resulted in different separate efficiencies. By increasing the methanol ratio in methanol–water (v/v) from 40% to 60%, the separation efficiency increased (Table 3). Thus, the eluent proportion was set as methanol‐water 6:4 (v/v).

**Table 3:** Purity and recovery of 3‐hydroxy‐4‐methoxy benzal acrolein with different mobile phase proportion

Mobile phase	3‐Hydroxy−4‐methoxy benzal acrolein	
	Purity(%)	Yield(%)
methanol/water(4:6, v/v)	80.80 ± 0.24	21.19 ± 0.25
methanol/water(5:5, v/v)	90.10 ± 0.10	25.08 ± 0.13
methanol/water(6:4, v/v)	98.56 ± 0.37	35.23 ± 0.22
methanol/water(7:3, v/v)	91.56 ± 0.37	31.23 ± 0.22

Note

The flow rate was 6 ml/min, the injected volume was 5 ml and sample concentration was 1 mg/ml.

To sum it up, the separation efficiency varied with changes in the mobile phase proportion, the flow rate and the loading amount. The optimum elution conditions were selected as the mobile phase ratio of methanol to water of 6:4 (v/v), the selected flow rate of 6 mg/ml and the loading amount of 10 mg.

#### 3.1.2 The absorption spectrum of 3‐hydroxy‐4‐methoxy benzal acrolein

Benzene ring have strong UV adsorption in the range of 180–184 nm and 200–204 nm, called E_1_ and E_2_ band respectively. And weak UV adsorption in the range of 230–270 nm called B band. When non‐bonded electron pairs of auxochromes (‐OH, ‐OR) connect with chromophore benzene ring, then producing Pπ‐conjugation, the absorption peak of the benzene ring moves to longer wavelength (Chernia & Gill, 1999). The absorption of carbonyl group is generally in the range of 200–205 nm, and the absorption band is significantly enhanced when the molecule contains double bonds conjugated with carbonyl groups (Marchal, Abdessalem, Tayakoutfayolle, & Uzio, 2010).

**Figure 1:**
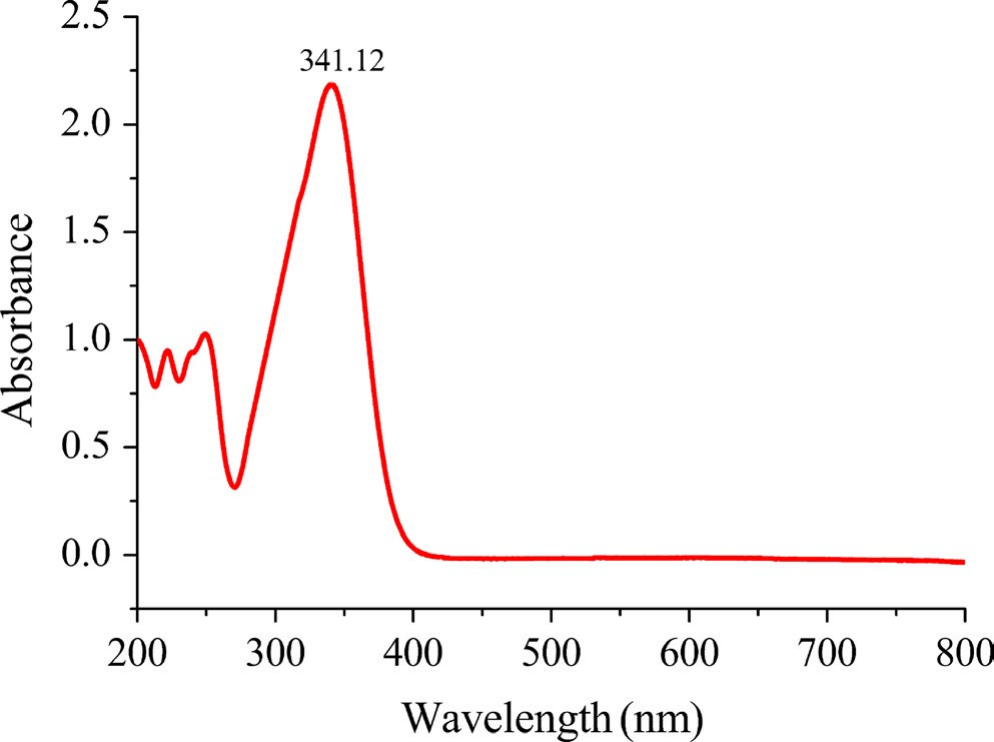
Ultraviolet scanning curve of 3‐hydroxy‐4‐methoxy benzal acrolein

The UV spectra of the purified intermediate characterized as 3‐hydroxy‐4‐methoxy benzal acrolein was shown in Figure 1. According to the aforesaid words the 218 nm and 248 nm should be the E_2_ band and B band of the benzene ring. The maximum absorbance wavelength of 3‐hydroxy‐4‐methoxy benzal acrolein was at 341 nm because of the Pπ‐conjugation between aldehyde group double bonds. So, the following experiment selected 341 nm as measured wavelength.

### 3.2 HPLC analysis

**Figure 2:**
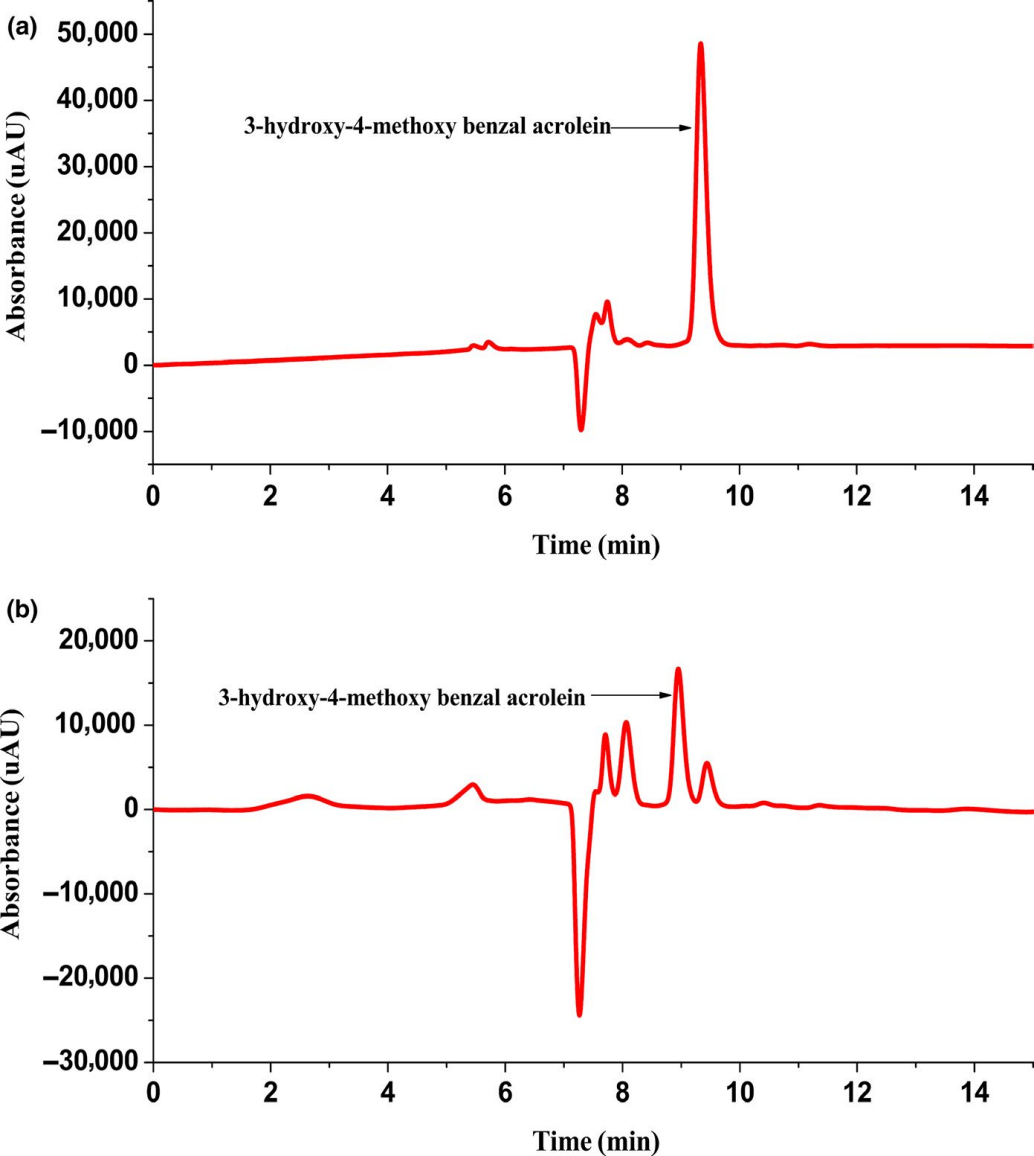
HPLC chromatogram of 3‐hydroxy‐4‐methoxy benzal acrolein reference substance (a) and sample(b)

Figure 2(a) is the chromatogram of intermediate reference substance, Figure 2(b) is the chromatogram of intermediate crude substance. Both graphs showed a solvent peak between 7.0 to 7.5 min. From Figure 2(a), it can be seen that the purified intermediate detected by HPLC only appeared a single peak, no impurity almost, while Figure 2(b) observed several impurity peaks at 7.5 to 8.5 min and 9.2 to 9.7 min, which might result from the unreacted raw materials and other by‐products. This indicated that it is well separated from the by‐product after the purification of column chromatography. The retention time of objective reference intermediate was 9.35 min and the content was 98.56% by area normalization method.

### 3.3 FTIR spectrum analysis

**Figure 3:**
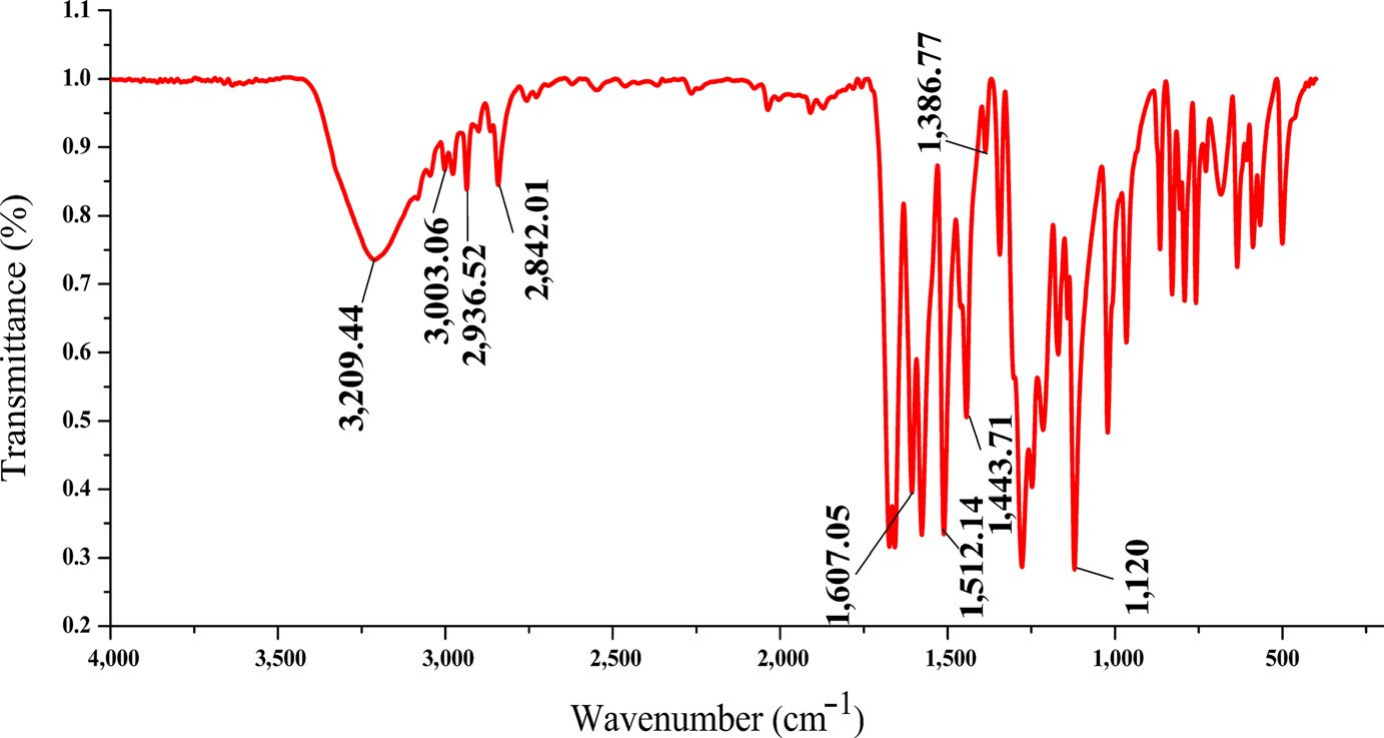
IR spectra of 3‐hydroxy‐4‐methoxy benzal acrolein

The skeleton stretching vibration of benzene ring, pyridine ring and other heterocyclic aromatic hydrocarbons is in the range of 1600–1400 cm^‐1^ (Zieba‐Palus and Kunicki, 2006), as shown in Figure 3, the peaks around 1607, 1512 and 1,443 cm^‐1^ might be caused by the benzene skeleton vibrations of 3‐hydroxy‐4‐methoxy benzal acrolein. The absorption peak at 3,003 cm^‐1^ is the absorption of the C‐H stretching vibrations of the benzene ring. So it is further proved that benzene ring exists in the molecule.

The FTIR spectra show a significant C = C bond peak at the region of 1680–1640 cm^‐1^ in the intermediate. The intensity of the absorption at 2,842 cm^‐1^ is the typical absorption of aldehyde group, suggesting the existence of –CHO group. The peak in 3,209 cm^‐1^ represents the vibration of hydroxyl groups in the benzene ring as the peak shape of hydrogen bonds is broad and blunt (Karabacak & Cinar, 2012). The absorption at 2,936 cm^‐1^ should be the stretching vibration of saturated C‐H bond in methoxyl group, and the absorption peak at 1,386 cm^‐1^ is due to the electronegativity of oxygen atoms in methoxy group, leading to the shift of the bending vibration of C‐H to a higher wave number, other than usually at 1000–1350 cm^‐1^ (Talari, Martinez, Movasaghi, Rehman, & Rehman, 2016), further proving that the ‐OCH_3_ partition is exited in the compound. Therefore, the signal characteristics of the infrared spectra consistent with the chemical structure as 3‐hydroxy‐4‐methoxy benzal acrolein.

### 3.4 MS analysis

**Figure 4:**
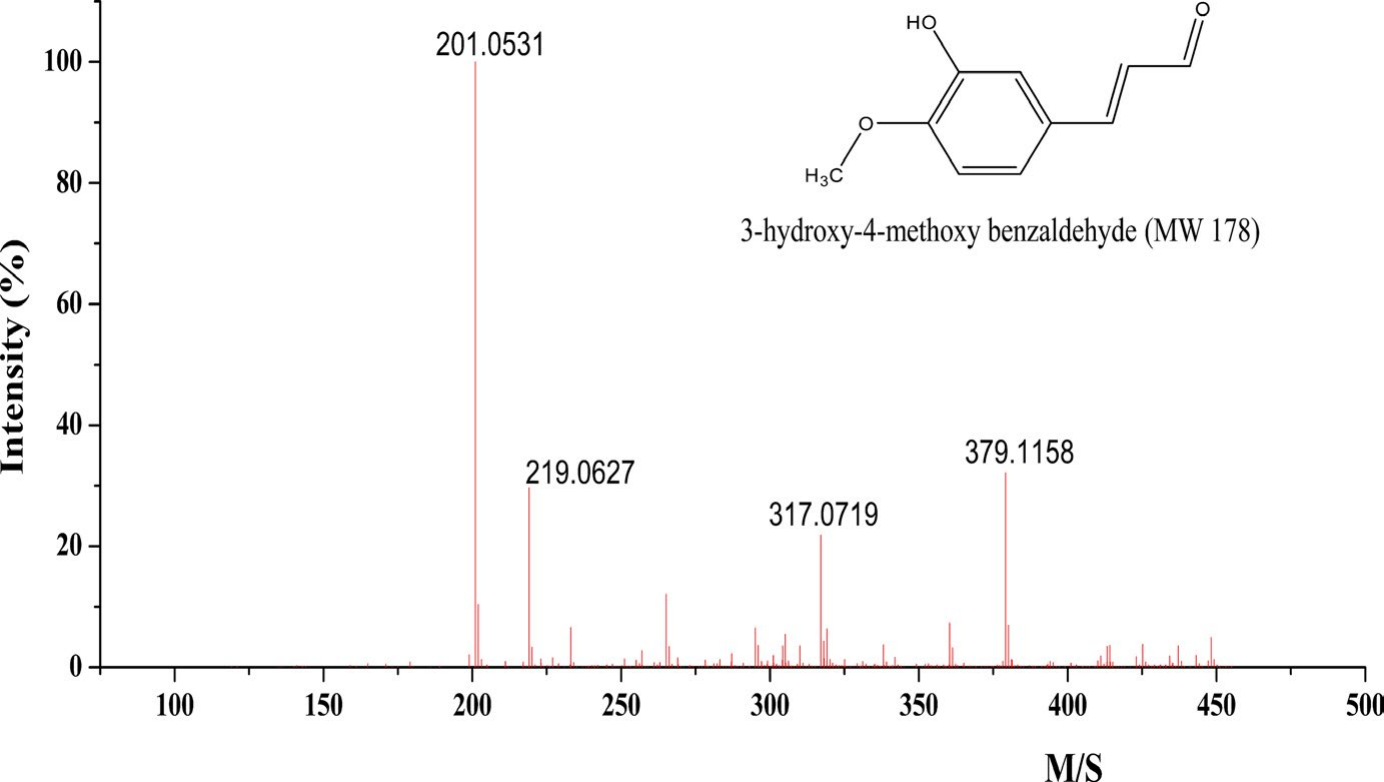
Mass spectrum of 3‐hydroxy‐4‐methoxy benzal acrolein

The mass spectral analysis of the purified intermediate was determined on the basis of high resolution mass spectrometry and B/E linked scan spectra (Figure 4). The relative molecular weight of the intermediate is 178. Results showed that the characteristic peak of [M + Na]^+^ at m/z 201.0531 was the base peak. The ionic peak of [2M‐K]^+^ was at m/z = 317.0719, which was the potassium ion peak of double molecular polymer. The peak at m/z = 379.1158 is the [2M + Na]^+^ ionic peak, which was the sodium ion peak of double molecular polymer. The reason for producing binary copolymer fractions might be the intermediate contains aldehyde groups causing the molecular polymerization (Cuyckens & Claeys, 2010).

In the positive ionization mode, single fraction peaks of the intermediate molecular connected with Na^+^ or K^+^ were observed, while proton peaks of the intermediate connected with H^+^ were not observed, due to the existence of a lot of sodium elements in the mass spectrometry system or in the mobile phase (Russell & Edmondson, 2015).

### 3.5 NMR analysis

**Figure 5:**
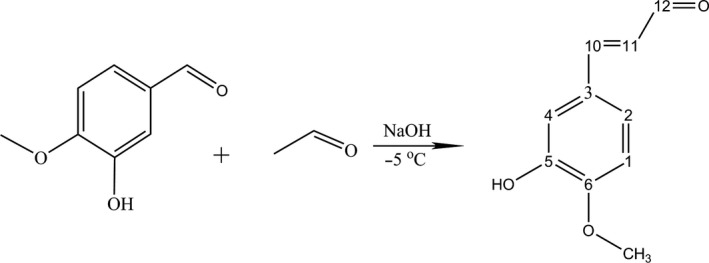
The process of preparation 3‐hydroxy‐4 methoxy benzal acrolein

**Figure 6:**
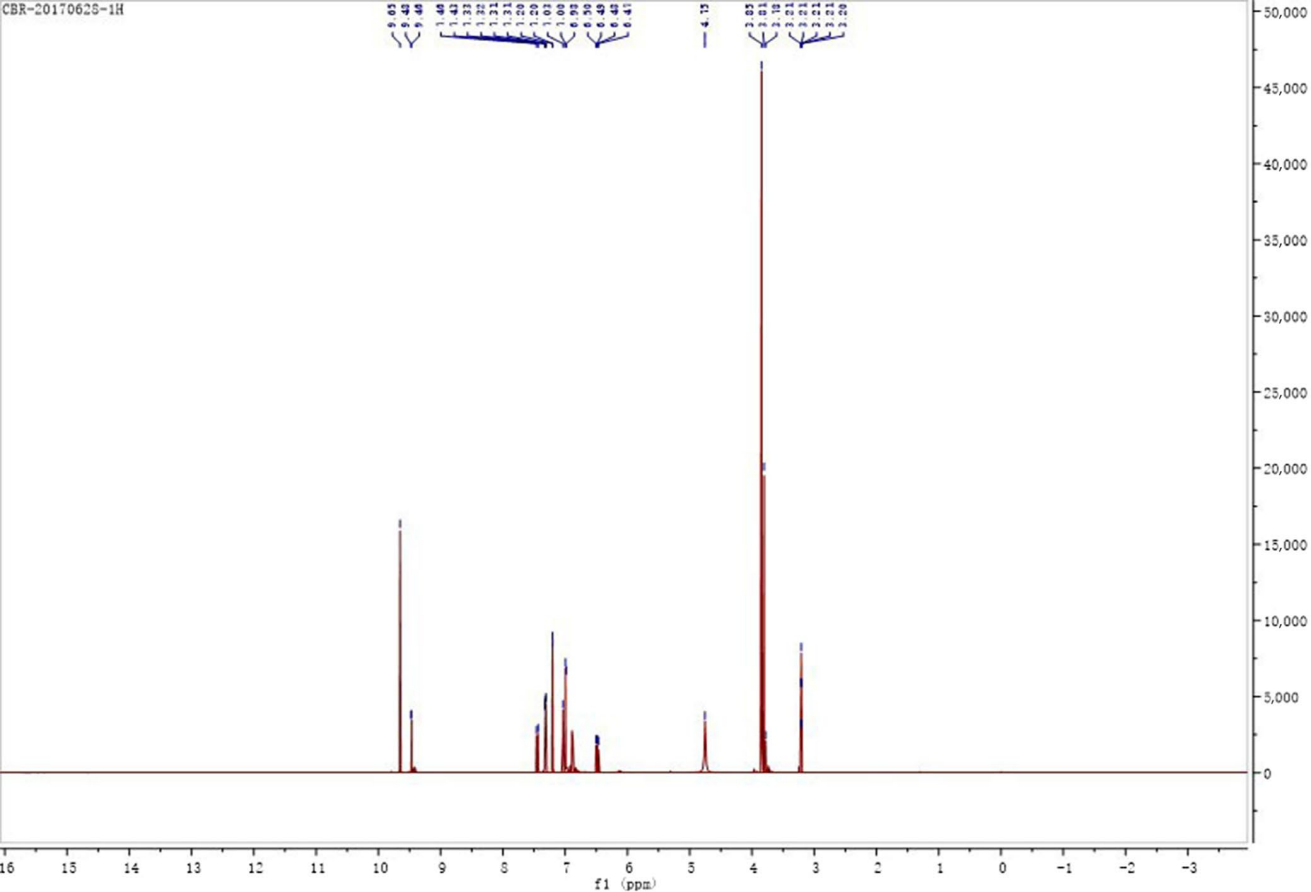
1H‐NMR spectrum of 3‐hydroxy‐4‐methoxy benzal acrolein

**Figure 7:**
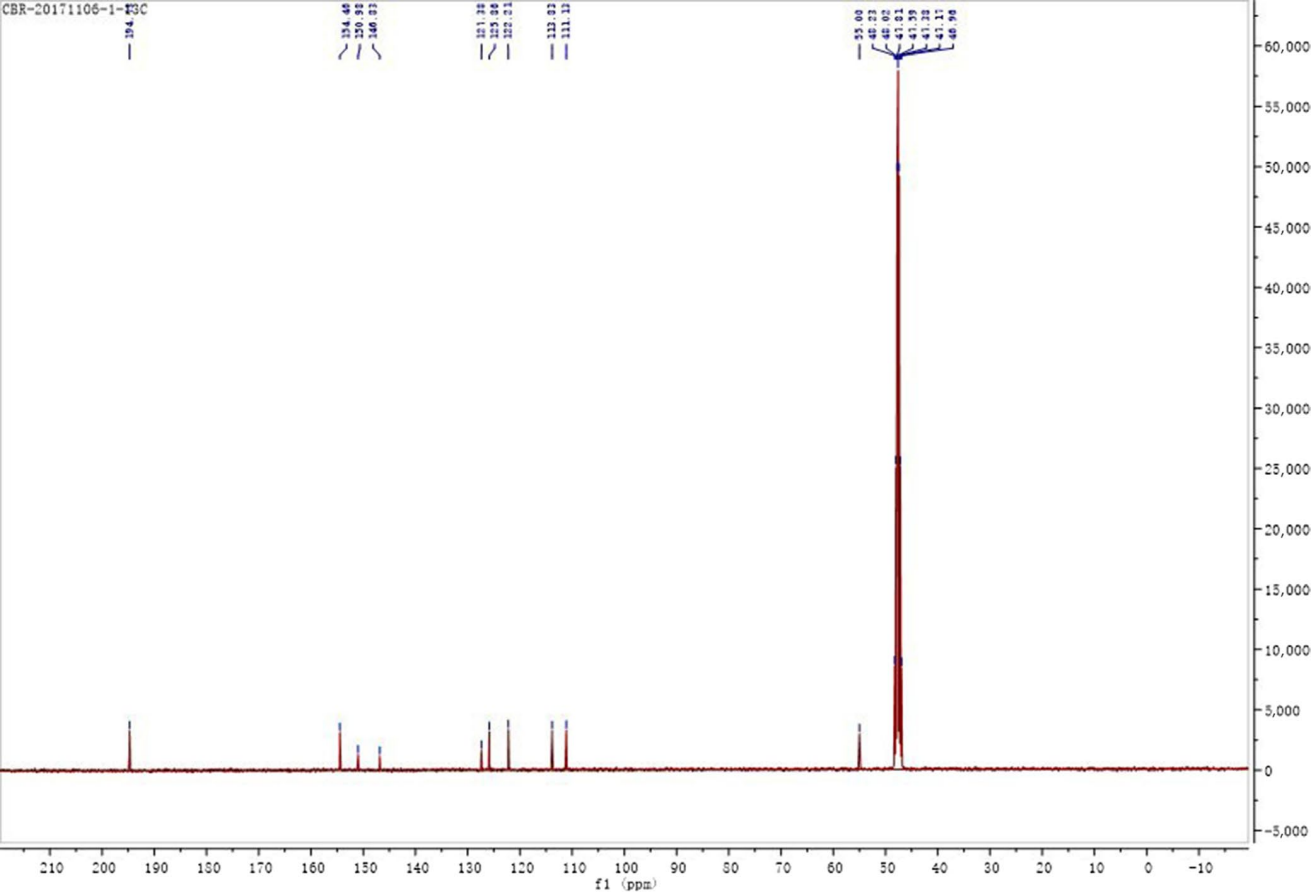
13C‐NMR spectrum of 3‐hydroxy‐4‐methoxy benzal acrolein

The structure of the intermediate was given in Figure 5. The ^1^H NMR spectrum and ^13^C‐NMR spectrum of the intermediate were shown in Figure 6 and Figure 7 respectively. The results of NMR analysis for the intermediate were as follows:

^1^H NMR (400 MHz, MeOD) δ: 9.65 (s, 1H, H‐12), 9.47 (d, 1H, H‐7), 7.19–7.08 (m, 2H, H‐10, H‐11), 7.32 (d, 1H, H‐4), 7.03 (s, 1H, H‐2), 6.99 (d, 1H, H‐1), 3.85 (s, 3H, H‐9). ^13^C NMR (400 MHz, MeOD) δ: 194.73 (C‐12), 154.46 (C‐5), 150.98 (C‐3), 146.83 (C‐6), 127.38 (C‐11), 125.86 (C‐10), 122.21 (C‐2), 113.83 (C‐1), 111.13 (C‐4), 55.00 (C‐9).

δ 7.32 (d, 1H), 7.03 (s, 1H), 6.99 (d, 1H) indicated the intermediate contained a 1, 3, 4 ‐ three substituted benzene ring in the structure. δ 7.19–7.08 (m, 2H) was the proton signal of the ‐CH = CH‐ structure. The ^13^C‐NMR spectra of the intermediate showed that it has 10 carbon atoms.

In total, all spectra data from FTIR, EI‐MS, ^1^H NMR and ^13^C NMR spectrum confirmed that the chemical structure of the sweetener intermediate synthesized in this study was 3‐hydroxy‐4‐methoxy benzal acrolein, and the HPLC detection showed the purity of the reference substance could be used for quantitative analysis.

### 3.6 Method validation of HPLC

In the chemical synthesis and quality control of intermediate products, HPLC is widely used because of its availability and effectiveness.

The standard curve was implemented by mean of determining the reference substance of different concentrations, and each concentration sample was tested 3 times. The concentration of the reference substance versus corresponding peak area was linear in the range of 0.005–0.08 mg/ml. The linear equation was found to be y = 30383x−13.005 and the correlation coefficient (r) was greater than 0.999, the relative uncertainty is 2%, the minimum relative uncertainty is 1%, showing the validation of the standard curve.

The results of method repeatability were shown in Table 4. The RSDs of intermediate peak area were 1.92% and 1.97% at the two concentration levels, respectively, indicating the method presented suitable repeatability for quantitative analysis of intermediate within the range from 0.005 to 0.08 mg/ml.

**Table 4:** Repeatability test results(*n* = 6)

Concentration	Peak area						Average peak area	RSD(%)
	1	2	3	4	5	6		
0.03 mg/ml	890.23	878.42	916.23	869.34	873.674	892.784	886.78	1.92%
0.06 mg/ml	1804.97	1,810.45	1829.32	1765.98	1759.08	1,850.92	1803.45	1.97%

An added sample recovery was conducted to determine the accuracy of the method (Table 5). The recovery rate was in the range of 94.5% to 106.37%. So, the present method is efficient for the intermediate analysis.

**Table 5:** Recovery of 3‐hydroxy‐4‐methoxy benzal acrolein (*n* = 3)

Sample	Standard added(μg)	Detected(μg)	Recovery(%)	RSD(%)
3‐Hydroxy−4‐methoxy benzal acrolein	0.4	0.378 ± 0.028	94.5 ± 0.07	3.58
	1.6	1.702 ± 0.54	106.37 ± 0.33	1.87
	3.2	3.283 ± 0.92	102.59 ± 0.28	2.38
	4.8	4.672 ± 0.78	97.33 ± 0.16	1.08
	6.4	6.512 ± 0.61	101.75 ± 0.09	1.23

The estimation of the limit of detection or quantitation was done on the basis of the ICH guidelines (Abbas, Fayez, & Abdel, 2006). The LOD (limit of detection, S/*N* ≥ 3) and the LOQ (limit of quantification, S/*N* ≥ 10) were measured according to the response and the slope. The LOD and LOQ values were 5 ng/ml and 15 ng/ml respectively.

## 4 CONCLUSION

In this study, a new low‐cost method for the preparation and purification of a sweetner intermediate named 3‐hydroxy‐4‐methoxy benzal acrolein was described. The crude intermediate was purified by isocratic elution using a methanol‐water (6:4, v/v) mobile phase as eluent. Results showed that the purification strategy was robust and efficient. In addition, a novel RP‐HPLC method was developed to allows the quantification of 3‐hydroxy‐4‐methoxy benzal acrolein in one 15‐min‐long analytical run. Satisfactory repeatability, high recoveries, limit of quantitation, and limit of detection make it suitable for quality control of standardized sweetner intermediate in food industry, and for characterization of intermediate could further used in research on their bioactivities. Of course, the purifying method and analysis method could be a reference to develop other sweetener intermediates, and the bioactivity of 3‐hydroxy‐4‐methoxy benzal acrolein can be further researched because of larger superdelocalizability than isovanillin. Therefore, the research work would be a great practical significance in advancing the process of industrialization of advantame.

## CONFLICTS OF INTEREST

All authors declare no conflict of interest.

## ETHICAL APPROVAL

The study did not involve any human or animal testing.
